# Evaluation of feed value of a by-product of pickled radish for ruminants: analyses of nutrient composition, storage stability, and in vitro ruminal fermentation

**DOI:** 10.1186/s40781-016-0117-1

**Published:** 2016-09-16

**Authors:** Seoyoung Jeon, Keun-Nam Sohn, Seongwon Seo

**Affiliations:** Division of Animal & Dairy Sciences, Chungnam National University, 99 Daehak-ro, Yuseong-gu, Daejeon, Republic of Korea

**Keywords:** By-product of pickled radish, In vitro fermentation, Nutrient composition, Ruminant feed, Storage stability

## Abstract

**Background:**

By-products of pickled radish (BPR) are considered food waste. Approximately 300 g/kg of the total mass of raw materials becomes BPR. Production of pickled radish has grown continuously and is presently about 40,000 metric tons annually in Korea. The objective of the present study was thus to explore the possibility of using BPR as a ruminant feed ingredient.

**Results:**

BPR contained a large amount of moisture (more than 800 g/kg) and ash, and comprised mostly sodium (103 g/kg DM) and chloride (142 g/kg DM). On a dry matter basis, the crude protein (CP) and ether extract (EE) levels in BPR were 75 g/kg and 7 g/kg, respectively. The total digestible nutrient (TDN) level was 527 g/kg and the major portion of digestible nutrients was carbohydrate; 88 % organic matter (OM) was carbohydrate and 65 % of total carbohydrate was soluble or degradable fiber. The coefficient of variation (CV) of nutrient contents among production batches ranged from 4.65 to 33.83 %. The smallest CV was observed in OM, and the largest, in EE. The variation in CP content was relatively small (10.11 %). The storage stability test revealed that storage of BPR at 20 °C (room temperature) might not cause spoilage for 4 d, and possibly longer. If BPR is refrigerated, spoilage can be deferred for 21 d and longer. The in vitro ruminal fermentation study showed that substitution of annual ryegrass straw with BPR improved ruminal fermentation, as evidenced by an increase in VFA concentration, DM degradability, and total gas production.

**Conclusion:**

The major portion of nutrients in BPR is soluble or degradable fiber that can be easily fermented in the rumen without adverse effects, to provide energy to ruminant animals. Although its high sodium chloride content needs to be considered when formulating a ration, BPR can be successfully used as a feed ingredient in a ruminant diet, particularly if it is one component of a total mixed ration.

## Background

The use of by-products of food processing has been of great interest in the livestock industry. By-products are residues derived from the processing of raw materials to manufacture food products for sale. They usually contain some degree of digestible nutrients that can be used as livestock feed [1]. Most of these by-products however, are wasted. As the food industry is continuously being developed, the generation of food by-products has also increased. This results in economic losses and environmental challenges [2]. Thus, converting food by-products to valuable biomass has been an important issue in the scientific community for the last two decades [1].

In this regard, various by-products, mainly from agricultural and food processing, have been tested for their nutritive value as potential animal feeds [3]. However, there are several challenges associated with the use of food by-products as animal feeds. The nutrient composition of an animal feed needs to be consistent and predictable [3]. The nutrient composition of food by-products however, can vary significantly according to the processing methods and condition of the raw materials, as well as their initial nutrient content [4]. Another major issue associated with food by-products is their moisture content. Most food by-products have a moisture content of more than 600 g/kg [4]. High moisture levels can cause spoilage, which leads to a bio-security risk [3]. Therefore, to expand the use of food by-products as animal feed, the consistency of nutrient composition and storage stability of the by-products should also be considered.

Many by-products are generated from the manufacture of pickled radish in Korea. By-products of pickled radish (BPR) comprise approximately 300 g/kg of raw material and are generally wasted [5]. The market for pickled radish has grown rapidly and total production is estimated to be about 40,000 metric tons annually [5]. One manufacturer of pickled radish reportedly lost about 300 million Korean won in wasted BPR within a single year. Besides its significant generation, BPR contains digestible nutrients, such as crude protein (60 g/kg) and crude fat (30 g/kg) [6]. In addition, various digestive enzymes (e.g., amylase, amidase, and glycosidase) contained in the raw material (*Raphanus sativus* L.) might remain in BPR and act as bio-active factors that can promote feed utilization [7]. However, to our knowledge, no study has been conducted to evaluate the feed value of BPR.

Therefore, the objective of the present study was to investigate the feed value of BPR as a ruminant feed ingredient. We tested the consistency of the nutrient composition of BPR and conducted fractionation and detailed chemical analysis. The storage stability of BPR at three different temperatures (i.e. 4 °C, 20 °C, and 37 °C) was determined. In addition, the effects of BPR supplementation on rumen fermentation were analyzed.

## Methods

### Sample preparation

The by-product of pickled radish, mixes with peels and whole wastes, was obtained from a manufacturer (Ilga, Sejong, Korea) three times at approximately 1-month intervals on April 22, May 27, and July 6, 2015. The BPR sample from the last batch was used to test storage stability. For each batch, BPR was thoroughly mixed, sampled, frozen at −80 °C, and freeze-dried at −50 °C for 96 h using a freeze dryer (VFD 0030-5085, IlShin, Dongducheon, Korea). The samples were then ground through a cyclone mill (Foss, Hillerød, Denmark) fitted with a 1 mm screen. The proximate nutrient composition of a sample of each batch was analyzed. Dry matter (DM, #930.15), crude protein (CP; #990.03), acid detergent fiber (ADF; #973.18), ether extract (EE; #920.39), and ash (#942.05) were determined as outlined by the AOAC [8]. Samples from all three batches were composited (equal weight, wet basis) and used for the in vitro fermentation study.

### Storage stability test

The BPR sample from the final batch was used to test storage stability. After being thoroughly mixed, BPR (1.5 ± 0.03 kg) was sampled in plastic (low-density polyethylene) bags (22 × 27 cm; Cleanwrap, Gimhae, Korea). The sample bags were randomly allotted to one of three groups in triplicate: low temperature (LT), room temperature (RT), and high temperature (HT). The LT bags were stored at 4 °C in a refrigerator (GC-114HCMP, LG, Seoul, Korea). The RT and HT bags were stored at 20 °C and 37 °C, respectively, in conventional temperature-controlled incubators (BI-1000 M, JEIO TECH., Daejeon, Korea). After being stored for 0, 2, 4, 7, 14, and 21 d, samples were taken from each bag and immediately stored at −80 °C until further analysis. After being thawed at 20 °C for 1 h, samples were filtered through 4-layer sterile gauze. The pH of the filtrate was measured using a pH meter (EcoMet P25, Istek Inc., Seoul, Korea) and the NH_3_-N concentration was determined [9].

### In vitro fermentation

The composited BPR sample, a commercial concentrate mix (CM) for Korean beef cows (Cargill Agri Purina Inc., Seongnam, Korea), and annual ryegrass straw (ARS) were used for the in vitro ruminal fermentation study. The control diet (CON) comprised 400 g/kg CM and 600 g/kg ARS. For the experimental diets, BPR replaced ARS at three levels: 150 g/kg (T15), 300 g/kg (T30), and 450 g/kg (T45); whereas the proportion of CM in the diet remained the same. Consequently, the proportion of BPR in the diet was 90 g/kg, 180 g/kg, and 270 g/kg in T15, T30, and T45, respectively. The nutrient composition of each feed and all experimental diets are presented in Tables 1 and 2.

**Table 1:** Chemical composition (g/kg DM or as stated) of feeds

Item^1^	BPR^2^	Ryegrass straw	Concentrate mix
DM, g/kg as fed	71	867	864
OM	709	944	938
CP	75	63	149
SOLP	36	20	48
NDICP	8	18	22
ADICP	3	18	15
aNDF	243	754	245
ADF	218	520	124
ADL	12	134	33
Ether extract	7	9	40
Ash	291	56	62
Ca	3	5	9
P	2	1	6
K	11	9	11
Na	103	4	4
Cl	142	7	3
S	3	2	3
TDN	527	429	766
NEm, MJ/kg DM	4.8	3.3	7.6
NEg, MJ/kg DM	2.4	1.1	5.0
Total carbohydrates	627	872	750
NFC	392	136	526
Carbohydrate fractions, g/kg carbohydrate			
CA	290	62	115
CB1	16	11	536
CB2	319	83	51
CB3	329	475	194
CC	45	369	104
Protein fractions, g/kg CP			
PA + B1	480	317	322
PB2	412	402	532
PB3	65	3	48
PC	43	278	98

^1^ *DM* dry matter, *OM* organic matter, *CP* crude protein, *SOLP* soluble CP, *NDICP* neutral detergent insoluble CP, *ADICP* acid detergent insoluble CP, *aNDF* neutral detergent fiber analyzed using a heat stable amylase and expressed inclusive of residual ash, *ADF* acid detergent fiber, *ADL* acid detergent lignin, *TDN* total digestible nutrients, *NEm* net energy for maintenance, *NEg* net energy for growth, *NFC* non-fiber carbohydrate, *CA* carbohydrate A fraction, ethanol soluble carbohydrates, *CB1* carbohydrate B1 fraction, starch, *CB2* carbohydrate B2 fraction, soluble fiber, *CB3* carbohydrate B3 fraction, available insoluble fiber, *CC* carbohydrate C fraction, unavailable carbohydrate, *PA + B1* protein A and B1 fractions, soluble CP, *PB2* protein B2 fraction, intermediate degradable CP, *PB3* protein B3 fraction, slowly degradable fiber-bound CP, *PC* protein C fraction, unavailable CP

^2^By-product of pickled radish. BPR was composited from three batches (equal weight, wet basis)

**Table 2:** Chemical composition (g/kg DM or as stated) of in vitro diets

	Treatment^2^			
Item^1^	CON	T15	T30	T45
DM, g/kg as fed	866	871	877	883
OM	942	920	899	878
CP	97	98	100	101
SOLP	31	33	34	36
NDICP	20	19	18	17
ADICP	17	15	14	13
aNDF	550	504	458	412
ADF	362	334	307	280
ADL	94	83	72	61
Ether extract	21	21	21	21
Ash	58	80	101	122
Ca	7	7	6	6
P	3	3	3	3
K	10	10	10	10
Na	4	13	22	31
Cl	5	18	30	42
Salinity	0	21	42	63

^1^ *DM* dry matter, *OM* organic matter, *CP* crude protein, *SOLP* soluble CP, *NDICP* neutral detergent insoluble CP, *ADICP* acid detergent insoluble CP, *aNDF* neutral detergent fiber analyzed using a heat stable amylase and expressed inclusive of residual ash, *ADF* acid detergent fiber, *ADL* acid detergent lignin

^2^CON: control diet consisting of 400 g/kg concentrate mix and 600 g/kg annual ryegrass straw; T15: 90 g/kg of annual ryegrass straw (15 %) was substituted with a by-product of pickled radish; T30: 180 g/kg of annual ryegrass straw (30 %) was substituted with a by-product of pickled radish; T45: 270 g/kg of annual ryegrass straw (45 %) was substituted with a by-product of pickled radish

Before feeding in the morning, rumen fluid was collected from three cannulated non-lactating Holstein cows that were fed a ration consisting of 550 g/kg ARS and 450 g/kg CM, twice daily at the Center for Animal Science Research, Chungnam National University, Korea. The study was approved by the Institutional Animal Care and Use Committee (IACUC) at Chungnam National University (IACUC approval No. CNU-00716).

Rumen contents from three cows were mixed in a glass bottle, placed on ice, and immediately transported to the laboratory. The rumen contents were strained through four layers of cheesecloth with glass wool and mixed with 4 × volumes of in vitro buffer solution [10] under strict anaerobic conditions. The rumen fluid/buffer mixture (50 mL) was transferred into 125 mL serum bottles containing 0.5 g of each experimental diet, under conditions of continuous flushing with O_2_-free CO_2_ gas. The bottles were sealed with butyl rubber stoppers and aluminum caps, and then incubated for 0, 3, 6, 12, 24, 48, and 72 h in an incubator at 39 °C.

After each incubation period, total gas production was measured using a pressure transducer (Sun Bee Instrument Inc., Seoul, Korea), as described by MK Theodorou, BA Williams, MS Dhanoa, AB McAllan and J France [11]. After the pH of the cultured fluid was measured, it was centrifuged at 21,000 × g for 10 min at 4 °C. The supernatant was used for analyses of volatile fatty acids (VFA) and ammonia nitrogen concentration. The remaining undegraded samples and fluid were used to determine in vitro ruminal DM degradability.

### Chemical analysis

The ARS and CM samples were dried at 65 °C for 72 h were ground through a cyclone mill (Foss, Hillerød, Denmark) fitted with a 1-mm screen. Further detailed chemical analysis of the ARS, CM and BPR samples was performed, based on the Cornell Net Carbohydrate and Protein System (CNCPS) fractionation scheme [12]. Nutrient composition was analyzed at Cumberland Valley Analytical Services Inc. (MD, USA). DM (#930.15), crude protein (#990.03), acid detergent fiber (#973.18), and ash (#942.05) were determined as outlined by the AOAC [13], and ether extract (#2003.05) was determined as described by the AOAC [14]. Crude protein was calculated as 6.25 times nitrogen content, and total nitrogen was measured by the Kjeldahl method, using a Leco FP-528 Nitrogen Combustion Analyzer (Leco, MI, USA). Acid detergent lignin (ADL) and neutral detergent fiber were analyzed using a heat stable amylase and expressed inclusive of residual ash (aNDF) as described by PJ Van Soest, JB Robertson and BA Lewis [15]. Soluble protein (SOLP) was determined as described by U Krishnamoorthy, TV Muscato, CJ Sniffen and PJ Van Soest [16]. The neutral detergent insoluble crude protein (NDICP) and acid detergent insoluble crude protein (ADICP) in each residue were also determined, as described by G Licitra, TM Hernandez and PJ Van Soest [17]. Ethanol soluble carbohydrate (ESC) and starch contents corrected for free glucose were analyzed as described by MB Hall [18], and mineral contents were determined using appropriate AOAC [19] methods. The total digestible nutrients (TDN), net energy for maintenance (NEm), and net energy for growth (NEg) were estimated based on equations of the NRC [20]. Non-fiber carbohydrates (NFC) were calculated as 100 - ash - EE - CP - (aNDF - NDICP) based on the guidelines of the NRC [20]. Dietary carbohydrate and protein fractions were estimated according to the CNCPS [12] with the following modifications. Sugars and organic acids (CA) were assumed to be equal to ESC; starch was denoted as CB1; and soluble fiber (CB2) was calculated as NFC - CA - CB1. Available NDF (CB3) was estimated by the equation (aNDF - NDICP) - (2.4 × ADL); and unavailable carbohydrate (CC) was estimated as 2.4 × ADL. For protein fractions, the sum of non-protein nitrogen and soluble true protein (PA + B1) was assumed to be equal to SOLP. Intermediate degradable CP (PB2) was estimated by the equation 100 - NDICP - SOLP. Slowly degradable fiber-bound CP (PB3) was estimated by the equation NDICP – ADICP; and unavailable CP (PC) was assumed to be equal to ADICP. All carbohydrate and protein fractions were expressed as g/kg of total carbohydrate and CP, respectively. Results of the analysis are presented in Table 1.

The NH_3_-N concentration was determined according to the methods of AL Chaney and EP Marbach [21]. Following re-centrifugation of BPR filtrate or in vitro cultured fluid at 21,000 × g for 15 min, 20 μL of the supernatant was mixed with 1 mL of phenol color reagent and 1 mL of alkali-hypochlorite reagent. The mixture was then incubated in a water bath for 15 min at 37 °C. After being mixed with 8 mL of distilled water, the optical density of the mixture was measured at 630 nm, using a spectrophotometer (UV-1800, Shimadzu, Japan).

The VFA concentration was determined as described by ES Erwin, GJ Marco and EM Emery [9]. In vitro cultured fluid (1 mL) was mixed with 0.2 mL of metaphosphoric acid (250 g/L) and kept at 4 °C for 30 min. Following centrifugation of the mixture at 21,000 × g for 10 min at 20 °C, the supernatant was injected into a gas chromatograph (HP 6890, Hewlett-Packard Co., CA, USA) equipped with a flame ionization detector (FID) and capillary column (Nukol™ Fused silica capillary column 30 m × 0.25 mm × 0.2 μm, Supelco Inc., PA, USA). The temperatures of the oven, injector, and detector were 90 °C–180 °C, 185 °C, and 210 °C, respectively. Nitrogen was used as the carrier gas at a flow rate of 40 mL/min.

### Statistical analysis

All data were analyzed using the MIXED procedure of SAS [22] with an appropriate statistical model for each analysis. In addition, comparisons were made between linear and quadratic functions among treatments for analysis of in vitro fermentation. Differences among treatments were also compared with the Tukey’s range test if there was a significant overall treatment effect. For the test of storage stability, repeated measures analysis was used to evaluate the effects of storage temperature. The interaction between treatment (i.e., different storage temperatures) and duration was of particular interest. Two-way ANOVA with fixed effects of hour of incubation, treatment, and interactions between those factors was used to analyze the in vitro ruminal fermentation characteristics. Linear and quadratic effects of the inclusion rate of BPR were tested using contrasts. Statistical significance was considered at *P* < 0.05, and a trend was determined at 0.05 ≤ *P* < 0.1.

## Results and discussion

The use of by-products of food processing has been of great interest in the livestock industry, particularly as it relates to reducing feed-related costs [23]. A significant amount of BPR is continuously being produced as the market for pickled radish keeps growing in Korea; however, most of it is wasted [5]. There is therefore solid justification to recycle BPR as a feed source for ruminant animals that have evolutionarily adapted to use low digestible nutrients efficiently in processes facilitated by symbiosis with rumen microbes [24].

To the best of our knowledge, this is the first study to explore the potential of BPR as an animal feed resource.

### Nutrient composition of the BPR

The BPR had high moisture content (more than 800 g/kg on an as fed basis, Table 1). Owing to a high level of ash (mostly sodium [103 g/kg DM] and chloride [142 g/kg DM], the organic matter content was 709 g/kg DM, which is lower than that of common feedstuffs (i.e., ARS and CM). On a dry matter basis, the CP and EE levels in BPR were 75 g/kg and 7 g/kg, respectively, which is comparable with those in ARS, whereas, the fiber content of BPR was just as low as that of CM. Cellulose comprised 85 % of the fiber content of BPR ([ADF - ADL]/NDF, Table 1). The TDN of BPR was 527 g/kg DM, which is between those of ARS and CM. The major portion of digestible nutrients was carbohydrate: 88 % of OM was carbohydrate and 62.5 % of total carbohydrate was NFC. NFC comprised mainly water-soluble carbohydrates (CA) and soluble fiber (CB2). Most of the CC represented a small proportion of aNDF (Table 1). A large proportion of CP in BPR was soluble (48 %) and only a small amount was fiber-bound (6.5 %), or unavailable (4.3 %) (Table 1). Besides sodium and chloride, the levels of other macro- or micro-minerals were low. Toxic micro-minerals (i.e., Hg, Cr, Pb, Cd, and As) were not detected in BPR.

A notable characteristic of BPR was its relatively high sodium chloride (NaCl) content. Ruminant animals are able to excrete large quantities of NaCl [25, 26]. Thus, they are tolerant of higher levels and increased intake of NaCl might not pose a problem if sufficient drinking water is provided [27, 28]. However, several studies have reported that high levels of NaCl negatively affects the growth of young sheep [29] and reduces growth rate and wool production in sheep, particularly when CP is also supplemented [30]. When balancing a ration with BPR, this higher NaCl content therefore needs to be considered.

The nutrient composition of BPR varied among the production batches. The coefficient of variation (CV) of nutrient contents ranged from 4.65 to 33.83 % (Table 3). The smallest CV was observed in OM, and the largest, in EE. The variation in CP content was relatively small (10.11 %); however, CVs in NDF, ADF, and ash were relatively large (22.49, 24.48, and 24.48 %, respectively). Among minerals, the CVs of Ca, P, and K were 18.08, 23.94, and 30.01 %, respectively. This indicated that variation among the mineral contents was also large.

**Table 3:** Variation in nutrient composition (g/kg DM or as stated) of by-product of pickled radish (BPR)

	BPR samples^2^					
Item^1^	Month 1	Month 2	Month 3	Mean	SD	CV
DM^3^, g/kg as fed	99.6	138.2	168.7	135.5	28.30	20.89
OM	666.8	735.0	739.3	713.7	33.21	4.65
CP	60.5	67.3	77.4	68.4	6.92	10.11
aNDF	216.3	369.8	365.3	317.2	71.32	22.49
ADF	170.6	312.7	297.5	260.3	63.71	24.48
Ether extract	7.8	10.7	17.5	12.0	4.05	33.83
Ash	333.2	265.0	260.7	286.3	63.71	24.48
Ca	4	3	3	3	0.61	18.08
P	2	1	1	1	0.33	23.94
K	16	9	9	11.3	3.40	30.01
Salinity	260	151	210	207.2	44.58	21.51

^1^ *DM* dry matter, *OM* organic matter, *CP* crude protein, *aNDF* neutral detergent fiber analyzed using a heat stable amylase and expressed inclusive of residual ash, *ADF* acid detergent fiber

^2^Samples were obtained three times on April 22, May 27, and July 6, 2015 from a pickled radish manufacturer (Ilga, Sejong, Korea)

^3^Dry matter content was based on the amount of residue after freeze-drying

Nutrient composition may vary according to the time and method of production. This can be an important issue when considering the use of food by-products as animal feeds [31]. Nevertheless, the variations in nutrient composition were within the range of wet by-products commonly used as feed ingredients. When the data provided by Dairy One Inc. [32] were analyzed, the average CVs among wet by-products were 33.0, 41.8, 43.8, 79.7, and 44.1 % for CP, NDF, ADF, EE, and ash, respectively; this feed composition library accessed in March, 2016. The wet by-products included apple pomace, beet pulp, carrot, citrus pulp, other fruit byproducts, and grape pomace, etc. High variability is commonly observed in the nutrient composition of feedstuffs; thus, the variations observed in BPR might not pose a major problem.

### Storage stability

**Fig. 1:**
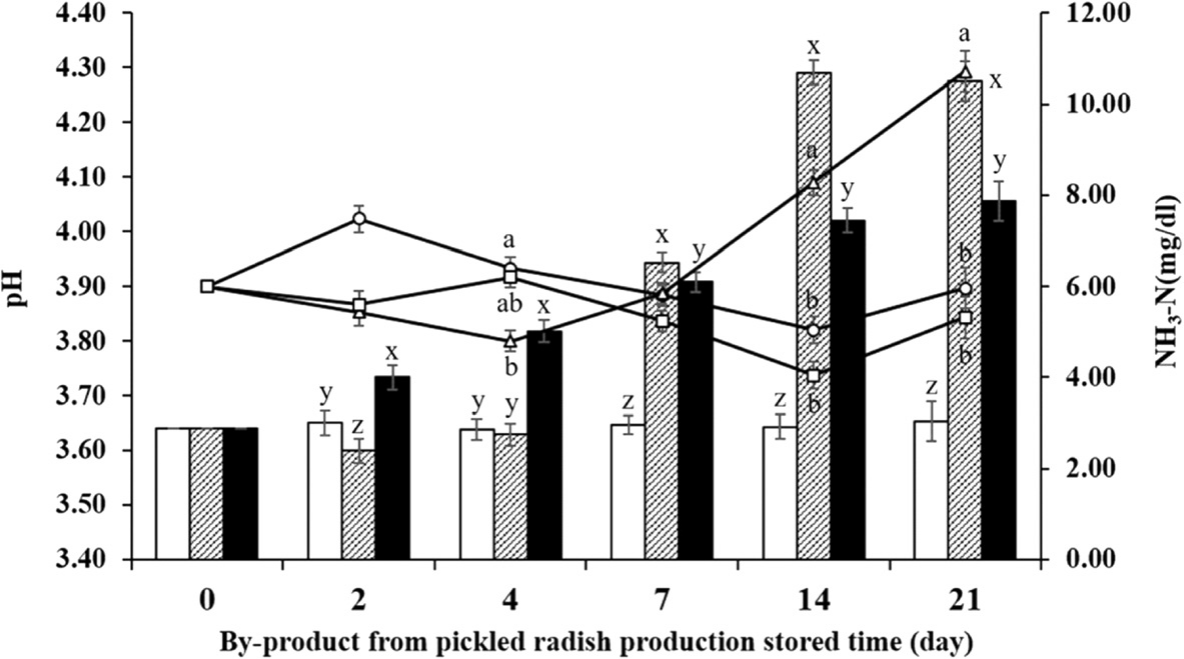
Changes in pH and ammonia nitrogen (NH_3_-N) concentration during storage at three different temperatures. The pH at low (4 °C, ○); room (20 °C, △); and high (37 °C, □) temperatures and the NH_3_-N concentrations at low (4 °C, open bar); room (20 °C, shaded bar); and high (37 °C, solid bar) temperatures. Each error bar represents the standard error of the mean temperature on each day. ^a-c^Means of pH that do not have common superscript differ (*P* < 0.05). ^x-z^Means of NH_3_-N concentration that do not have common superscript differ (*P* < 0.05)

Significant effects of temperature, storage duration, and the interactions between those two factors were noted on pH and NH_3_-N concentration (*P* < 0.05, Fig. 1). The RT group yielded a significantly higher pH on days 14 and 21 in comparison to the other treatments (*P* < 0.05). The pH of the RT group declined until day 4, but increased thereafter. On the other hand, the pH of the LT group increased on day 2, was subsequently reduced until day 14, and increased again on day 21. The pH trend of the HT group was similar to that of the LT group. However, the highest pH in the HT group was observed on day 4, whereas that in the LT group, on day 2.

The NH_3_-N concentration in the LT group was not altered during storage; however, that in the RT and HT groups was significantly increased during storage (*P* < 0.05, Fig. 1). A sudden increase in NH_3_-N concentration was observed in the RT group on day 7. The NH_3_-N concentration in the RT group was significantly higher on days 14 and 21 in comparison to other groups (*P* < 0.01). The HT group yielded significantly higher NH_3_-N concentrations on days 2 and 4 (*P* < 0.01) that constantly increased thereafter, for the duration of storage.

Since BPR contains a large amount of moisture (more than 800 g/kg on an as fed basis), storage stability was an important consideration. Handling, storage, and feeding challenges can occur if a feed ingredient has a moisture level that is higher than 200 g/kg on an as fed basis [33]. One of the major issues with high moisture contents in food-by products is the potential for spoilage during storage and transport [4]. Since the survival of microbes depends on moisture levels [34], a high moisture content can cause spoilage, which leads to bio-security risks [3].

The results of the storage stability test revealed that despite its high moisture content, storage of BPR even at room temperature might not cause spoilage for 4 d and possibly later. If refrigerated, spoilage of BPR can be deferred until 21 d and possibly later. Owing to its high moisture content, BPR can be used as a feed ingredient in a total mixed ration (TMR). If this is the case, the possibility of spoilage can be further reduced by fermentation of the TMR [35].

### In vitro fermentation

As the level of substitution of ARS by BPR increased, in vitro ruminal pH was significantly reduced in a linear manner at each time point from 3 to 48 h (*P* < 0.01, Table 4). For the first 12 h of in vitro fermentation, NH_3_-N concentrations declined in a linear manner, as the proportion of BPR increased (Table 4). The NH_3_-N concentration of CON was highest at 12 h. The NH_3_-N concentrations at 12 and 24 h did not differ significantly among treatments. However, at 48 and 72 h, NH_3_-N concentrations increased in a linear manner, as inclusion of BPR increased. The NH_3_-N concentration of T45 was significantly higher than that of CON (*P* < 0.05). As BPR inclusion levels increased, total VFA increased in a linear manner up to 12 h and at 72 h (Table 4). At 6, 12, and 72 h, total VFA concentrations were significantly higher in T45 than in CON (P < 0.05). The acetate/propionate (A/P) ratio was higher in CON than in T45 (*P* < 0.01) for up to 12 h of in vitro ruminal fermentation; however, the A/P ratio of T45 became significantly higher than that of CON after 24 h (*P* < 0.01, Table 4). For up to 12 h, the A/P ratio declined in a linear manner, as BPR inclusion levels increased; however, after 24 h, this relationship became a linear decline with increasing levels of BPR.

**Table 4:** The pH and concentration of NH_3_-N and VFA after in vitro ruminal fermentation

	Treatment^1^					*P*-value		
Item	CON	T15	T30	T45	SEM	Mean	Linear	Quadratic
pH								
3 h	6.58^a^	6.56^ab^	6.55^b^	6.55^b^	0.004	0.01	<0.01	0.08
6 h	6.56^a^	6.54^ab^	6.54^bc^	6.52^c^	0.003	<0.01	<0.01	0.35
12 h	6.51^a^	6.48^b^	6.45^c^	6.43^d^	0.003	<0.01	<0.01	0.63
24 h	6.44	6.43	6.41	6.41	0.006	0.02	<0.01	0.81
48 h	6.39^a^	6.38^ab^	6.36^b^	6.27^b^	0.005	0.02	<0.01	0.09
72 h	6.40	6.40	6.40	6.40	0.005	0.75	0.89	0.75
NH_3_-N (mg/dL)								
3 h	10.97	10.02	9.41	8.80	0.662	0.20	0.04	0.81
6 h	12.95^a^	12.76^ab^	12.24^ab^	11.80^b^	0.243	0.04	<0.01	0.63
12 h	12.95	12.18	11.27	11.36	0.713	0.36	0.11	0.56
24 h	15.22	15.32	16.15	15.49	0.504	0.59	0.49	0.48
48 h	20.69^b^	22.54^a^	22.28^a^	23.77^a^	0.624	0.05	0.01	0.78
72 h	27.12^b^	26.68^b^	29.53^a^	30.40^a^	0.352	<0.01	<0.01	0.10
Total VFA (mM)								
3 h	19.73	20.24	20.57	21.16	0.491	0.29	0.07	0.94
6 h	23.32^b^	24.58^ab^	28.20^a^	27.85^a^	0.872	<0.01	<0.01	0.38
12 h	31.54^c^	34.39^bc^	36.90^ab^	38.11^a^	0.668	<0.01	<0.01	0.25
24 h	40.11	46.47	46.97	43.57	1.751	0.08	0.20	0.02
48 h	51.08	53.99	53.61	51.93	2.255	0.77	0.83	0.34
72 h	53.14^b^	55.06^b^	58.42^a^	60.17^a^	0.570	<0.01	<0.01	0.89
A:P ratio								
3 h	4.07^a^	3.97^b^	3.87^c^	3.83^c^	0.017	<0.01	<0.01	0.16
6 h	4.07^a^	4.06^a^	3.95^b^	3.96^b^	0.014	<0.01	<0.01	0.46
12 h	4.15^a^	4.13^a^	3.95^b^	3.94^b^	0.013	<0.01	<0.01	0.60
24 h	3.77^c^	3.83^bc^	3.88^ab^	3.96^a^	0.019	<0.01	<0.01	0.58
48 h	3.64^c^	3.72^bc^	3.79^ab^	3.85^a^	0.023	<0.01	<0.01	0.52
72 h	3.60^c^	3.67^b^	3.73^a^	3.77^a^	0.010	<0.01	<0.01	0.15

^1^CON: Control diet consisting of 400 g/kg concentrate mix and 600 g/kg annual ryegrass straw; T15: 90 g/kg of annual ryegrass straw (15 %) was substituted with a by-product of pickled radish; T30: 180 g/kg of annual ryegrass straw (30 %) was substituted with a by-product of pickled radish; T45: 270 g/kg of annual ryegrass straw (45 %) was substituted with a by-product of pickled radish

^a-d^Means that do not have common superscript differ (*P* < 0.05)

Significant linear and quadratic relationships were observed in in vitro ruminal DM degradability (IVDMD), with increasing BPR inclusion levels at most time points (Table 5). The IVDMD of T15 was not significantly different from that of CON at all time points; however, the IVDMD of T30 was greater than that of CON throughout the incubation period, except at 48 h. The IVDMD of T45 was significantly higher than that of the other treatments at all time points (*P* < 0.05). The IVDMD did not differ significantly between T15 and T30, except at 24 h. The total gas production significantly increased in a linear manner throughout the process of in vitro fermentation, as BPR inclusion levels increased (Table 6). No significant differences were noted in total gas production between T30 and T45 at any time point.

**Table 5:** Effect of by-product of pickled radish on in vitro ruminal DM degradability (IVDMD, g/kg DM)

	Treatment^1^					*P*-value		
Time	CON	T15	T30	T45	SEM	Mean	Linear	Quadratic
6 h	63.4^c^	75.4^bc^	108.1^b^	204.9^a^	9.05	<0.01	<0.01	<0.01
12 h	143.6^c^	180.0^bc^	234.2^b^	321.7^a^	13.50	<0.01	<0.01	0.09
24 h	312.8^c^	336.3^c^	381.1^b^	492.3^a^	4.39	<0.01	<0.01	<0.01
48 h	477.3^d^	491.7^b^	534.9^b^	630.4^a^	13.72	<0.01	<0.01	0.02
72 h	556.0^a^	577.9^bc^	589.8^b^	682.7^a^	5.22	<0.01	<0.01	<0.01

^1^CON: Control diet consisting of 400 g/kg concentrate mix and 600 g/kg annual ryegrass straw; T15: 90 g/kg of annual ryegrass straw (15 %) was substituted with a by-product of pickled radish; T30: 180 g/kg of annual ryegrass straw (30 %) was substituted with a by-product of pickled radish; T45: 270 g/kg of annual ryegrass straw (45 %) was substituted with a by-product of pickled radish

^a-c^Means that do not have common superscript differ (*P* < 0.05)

**Table 6:** Effect of by-products of pickled radish on in vitro ruminal total gas production (mL)

	Treatment^1^					*P*-value		
Time	CON	T15	T30	T45	SEM	Mean	Linear	Quadratic
3 h	24.97	26.23	27.17	27.19	0.515	0.05	0.01	0.26
6 h	45.91^b^	48.84^ab^	51.81^a^	53.70^a^	1.184	<0.01	<0.01	0.67
12 h	70.17^c^	74.02^b^	86.14^a^	86.09^a^	0.749	<0.01	<0.01	0.03
24 h	101.03^c^	106.71^bc^	115.02^ab^	118.69^a^	1.941	<0.01	<0.01	0.62
48 h	116.75^b^	132.23^ab^	137.18^a^	143.15^a^	4.467	0.02	<0.01	0.32
72 h	146.02^b^	150.49^ab^	152.63^ab^	158.54^a^	0.345	0.03	<0.01	0.77

^1^CON: Control diet consisting of 400 g/kg concentrate mix and 600 g/kg annual ryegrass straw; T15: 90 g/kg of annual ryegrass straw (15 %) was substituted with a by-product of pickled radish; T30: 180 g/kg of annual ryegrass straw (30 %) was substituted with a by-product of pickled radish; T45: 270 g/kg of annual ryegrass straw (45 %) was substituted with a by-product of pickled radish

^a-c^Means that do not have common superscript differ (*P* < 0.05)

In vitro ruminal incubation using strained rumen fluid is a very useful technique to assess ruminal fermentation in vivo [36, 37]. The VFA concentrations of the cultured fluid, DM degradability, and total gas production after in vitro ruminal fermentation can be good indicators to evaluate the nutritional value of novel feed ingredients [38]. The present study demonstrated that substitution of ARS with BPR improves ruminal fermentation, as evidenced by increased VFA concentration, DM degradability, and total gas production. Since the A/P ratio did not decline with a longer incubation time, the acid load from rapid fermentation that could lead to metabolic disorders [39] might not pose a problem with BPR supplementation. This could be attributed to the fact that approximately 65 % of the carbohydrate content of BPR is soluble fiber and degradable NDF (the sum of CB2 and CB3 fractions, Table 1), both of which are rapidly fermented in the rumen without the production of lactic acid [40]. Moreover, the high NaCl content in BPR did not adversely affect microbial fermentation in the rumen. Based on the results of the in vitro fermentation study, BPR can be successfully used as a feed ingredient in a ruminant diet.

## Conclusion

In conclusion, the major portion of nutrients in BPR comprises soluble or degradable fiber that can be easily fermented in the rumen without adverse effects, to provide energy to ruminant animals. Although its high NaCl content needs to be considered when formulating a ration, BPR can be effectively used as a feed ingredient in a ruminant diet, particularly if included as one component of a TMR.

## Abbreviations

A/P: Acetate/propionate
ADF: Acid detergent fiber
ADICP: Acid detergent insoluble crude protein
ADL: Acid detergent lignin
aNDF: neutral detergent fiber
ARS: Annual ryegrass straw
BPR: By-products of pickled radish
CM: Commercial concentrate mix
CNCPS: Cornell Net Carbohydrate and Protein System
CP: Crude protein
CV: Coefficient of variation
DM: Dry matter
EE: Ether extract
ESC: Ethanol soluble carbohydrate
HT: High temperature
IACUC: Institutional Animal Care and Use Committee
IVDMD: In vitro ruminal DM degradability
LT: Low temperature
NDICP: Neutral detergent insoluble crude protein
NEg: Net energy for growth
NEm: Net energy for maintenance
NFC: Non-fiber carbohydrates
OM: Organic matter
RT: Room temperature
TDN: Total digestible nutrient
TMR: Total mixed ration
